# Endpoint Distribution Modeling-Based Capture Algorithm for Interfering Multi-Target

**DOI:** 10.3390/s24248191

**Published:** 2024-12-22

**Authors:** Xiangliang Zhang, Junlin Li, Pengjie Li, Fang Si, Xiangzhi Liu, Yu Gu, Shuguang Meng, Jibin Yin, Tao Liu

**Affiliations:** 1 The State Key Laboratory of Fluid Power and Mechatronic Systems, School of Mechanical Engineering, Zhejiang University, Hangzhou 310027, China; xlzh@zju.edu.cn (X.Z.); liuxiangzhi@zju.edu.cn (X.L.); yu.gu@zju.edu.cn (Y.G.); 2 Beijing Research Institute of Mechanical and Electrical Engineering, Beijing 102200, China; li_junlin8899@outlook.com (J.L.); 18201013816@163.com (P.L.); sifang1108@163.com (F.S.); 3 Faculty of Information Engineering and Automation, Kunming University of Science and Technology, Kunming 650093, China; m192999123@163.com

**Keywords:** human–computer interaction, pointing interaction, target distribution model, target capture, remote controls, trajectory planning

## Abstract

In physical spaces, pointing interactions cannot rely on cursors, rays, or virtual hands for feedback as in virtual environments; users must rely solely on their perception and experience to capture targets. Currently, research on modeling target distribution for pointing interactions in physical space is relatively sparse. Area division is typically simplistic, and theoretical models are lacking. To address this issue, we propose two models for target distribution in physical space-pointing interactions: the single-target pointing endpoint distribution model (ST-PEDM) and the multi-target pointing endpoint distribution model (MT-PEDM). Based on these models, we have developed a basic region partitioning algorithm (BRPA) and an enhanced region partitioning algorithm (ERPA). We conducted experiments with 15 participants (11 males, and four females) to validate the proposed distribution models and region partitioning algorithm. The results indicate that these target distribution models accurately describe the distribution areas of targets, and the region partitioning algorithm demonstrates high precision and efficiency in determining user intentions during pointing interactions. At target distances of 200 cm and 300 cm, the accuracy without any algorithm is 60.54% and 42.39%, respectively. Using the BRPA algorithm, the accuracy is 72.94% and 68.57%, while, with the ERPA algorithm, the accuracy reaches 84.11% and 82.74%, respectively. This technology can be utilized in interaction scenarios involving handheld pointing devices, such as handheld remote controls. Additionally, it can be applied to the rapid capture control and trajectory planning of drone swarms. Users can quickly and accurately capture and control target drones using pointing interaction technology, issue commands, and transmit data through smart glasses, thereby achieving effective drone control and trajectory planning.

## 1. Introduction

In physical space, pointing interactions cannot rely on methods like cursors, rays, or virtual hands for pointing feedback as in virtual space. Instead, they must depend on the user’s senses and experience to capture targets. When users perform pointing-based target capture in physical space, their hands lack physical support [1], and physiological tremors of the body [2] hinder accurate target selection. During the process of pointing selection, users must also maintain the stability of the pointing device until confirmation of selection, such as pressing a button on a remote control or pulling a trigger. Such confirmation actions may alter the pointing direction, resulting in a deviation between the actual selected position and the target position, known as the Heisenberg effect [3,4]. The Heisenberg effect in human–computer interaction can have certain impacts. For example, hand-based pointing is susceptible to small tremors that cause deviations from the target [5]. Similarly, discrete inputs, such as pressing a button, can disrupt the position of the interaction device, leading to spatial Heisenberg effects and introducing errors during the interaction process [4]. When the target is far from the user, the stability of pointing selection is typically significantly affected [6]. Interfering targets in physical space can also influence the accuracy of user pointing and selection.

Currently, research on pointing interaction technology primarily focuses on the VR/AR domain. It involves using handheld controllers to emit lasers into virtual scenes and select the first object intersecting with the beam [7,8,9,10]. Due to its simple and efficient characteristics, many studies are based on this pointing interaction technology [11,12,13,14]. Research on pointing interaction technology in physical space is relatively limited, mostly focusing on display screens, graphical interfaces, and pointing interaction technologies with cursor-based interfaces [15,16,17,18,19,20].

Over the years, researchers have also explored the distribution of endpoint selections. Murata observed that the distributions of horizontal and vertical coordinates of endpoints approximate a Gaussian distribution [21]. Recently, Grossman et al. developed a probability model for two-dimensional rapid pointing tasks, showing that deviations in both directions increase with target distance [22]. Bi et al. studied the distribution of touch input endpoints and proposed a dual-distribution hypothesis, deriving FFitts’ law based on endpoint distribution to predict finger touch performance [23]. Yu et al. explored endpoint distribution models for pointing selections in VR environments, introducing the EDModel [11]. Wei et al. proposed a prediction of endpoint distribution based on the eye and head [24].

Currently, research on region partitioning algorithms in pointing interaction techniques is relatively sparse. Most pointing interaction methods initiate interaction only upon complete target selection. However, this approach requires targets to be strategically placed and may not adequately meet the distribution requirements for multiple targets. In complex pointing scenarios involving multiple targets, region partitioning algorithms often face issues with overlapping partitions. Therefore, there is a need to segment overlapping regions. Research in this area generally falls into two categories: region segmentation based on image recognition [25,26] and region partitioning based on Voronoi diagrams [26,27,28,29]. Voronoi diagrams can be applied in spatial distribution analysis [30,31,32], trajectory planning [33,34], and coverage in sensor networks [35]. Miah et al. proposed a region coverage optimization algorithm based on Voronoi diagrams [36]; Li et al. utilized Voronoi diagrams for area division of event pixels and neighborhood construction [37]; Li and Chen proposed a Voronoi-based method to solve the coverage problem of unknown deformable targets [38]; Afghantoloee et al. introduced a purpose-oriented 3D Voronoi algorithm for optimizing sensor deployment in complex environments [39].

**Figure 1:**
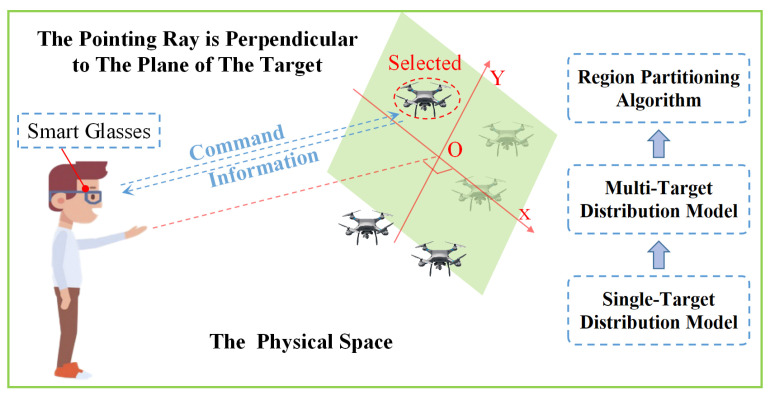
Application scenarios and proposed models. It can be applied to rapid capture control; users can quickly and accurately capture and control target drones using pointing interaction technology, issue commands, and transmit data through smart glasses.

In response to the aforementioned issues, we propose two target distribution models for pointing interactions in physical space: the single-target pointing endpoint distribution model (ST-PEDM) and the multi-target pointing endpoint distribution model (MT-PEDM). The ST-PEDM predicts the distribution of endpoint tasks for pointing selections based on target distance, visual error, and the Heisenberg effect. The MT-PEDM can predict the distribution areas of endpoint tasks for pointing selections in complex multi-target scenarios. Based on these two models, we propose a basic region partitioning algorithm (BRPA) and an enhanced region partitioning algorithm (ERPA). These algorithms exhibit high accuracy and efficiency, enabling precise determination of user intentions during pointing interactions. It can be applied to the rapid capture control and trajectory planning of drone swarms. Users can quickly and accurately capture and control target drones using pointing interaction technology, issue commands, and transmit data through smart glasses, thereby achieving effective drone control and trajectory planning, as shown in Figure 1.

## 2. Models and Algorithms

The endpoint refers to the coordinates where the directional ray emitted from a handheld pointing device intersects with the plane of the target control device during user interaction. These endpoints are typically distributed around the target in a scattered pattern.

### 2.1. Single-Target Pointing Endpoint Distribution Model

The pointing selection task is divided into two stages: pointing and confirmation of selection. Previous studies [7,40] have shown that, during the pointing stage, factors such as directional tilt and physiological tremors lead to variability in endpoint locations, resulting in a two-dimensional normal distribution of endpoints. The discrepancy between actual and perceived endpoints due to inconsistent gaze and pointing directions affects the expected endpoint distribution model μ. The probability density function of the two-dimensional normal distribution of pointing endpoints is given by Equation (1), where *p* is the correlation coefficient between *x* and *y*, and  $\mu_{x}$, $\mu_{y}$, $\sigma_{x}$, and  $\sigma_{y}$ are the mean and standard deviations of the distributions of variables *x* and *y*, respectively.

(1) $$\left\{\begin{array}{cc} \; & f\left(x,y\right)={\left(A\right)}^{2}exp\left[-\frac{1}{2\left(1-\rho^{2}\right)}\;\left(B+C\right)\right]\\ \; & A=2\pi \sigma_{x}\sigma_{y}\sqrt{1-\rho^{2}}\\ \; & B=\frac{{\left(x-\mu_{x}\right)}^{2}}{\sigma_{x}^{2}}-\frac{2\rho \left(x-\mu_{x}\right)\left(y-\mu_{x}\right)}{\sigma_{x}\sigma_{y}}\\ \; & C=\frac{{\left(y-\mu_{y}\right)}^{2}}{\sigma_{y}^{2}} \end{array}\right.$$

The selection confirmation stage is also influenced by the Heisenberg effect, impacting the distribution of endpoints. The target distance *D*, the Heisenberg effect, and their interactions affect the parameters of the two-dimensional average model for endpoint distribution, including the expectation μ and the covariance matrix ∑, as shown in Equation (2).

(2) $$\left\{\begin{array}{cc} \; & X∼\;N_{2}\left(\mu ,\Sigma \right)\;\;\;\;\;\mu =\left[\begin{array}{c} \mu_{x}\\ \mu_{y} \end{array}\right]\\ \; & \Sigma =[\begin{array}{cc} \sigma_{x}^{2} & \rho \sigma_{x}\sigma_{y}\\ \rho \sigma_{x}\sigma_{y} & \sigma_{y}^{2} \end{array}] \end{array}\right.$$

When engaging in directional interactive operations, as the target distance *D* increases, the influence of selection error and physiological tremor on the range of endpoint distribution is magnified, thereby affecting the covariance matrix ∑. The impact of visual error interaction on the offset of the expectation µ of the endpoint distribution model is also amplified. Based on this, we propose an endpoint distribution model without the Heisenberg effect, as shown in Equation (3), where constants a,b,c,d,e,f,g, and  *h* are determined empirically.

(3) $$\begin{array}{c} \mu =\left[\begin{array}{c} a+bD\\ c+dD \end{array}\right]\;\;\;\;\Sigma =\left[\begin{array}{cc} {\left(e+fD\right)}^{2} & 0\\ 0 & {\left(g+hD\right)}^{2} \end{array}\right] \end{array}$$

The single-target endpoint distribution model with the Heisenberg effect is shown in Equation (4), where constants b, c, d, m, n, o, p, q, and r are determined empirically.

(4) $$\begin{array}{c} \mu =\left[\begin{array}{c} m+(b+n)D\\ c+dD \end{array}\right]\;\;\Sigma =\left[\begin{array}{cc} {\left(o+pD\right)}^{2} & 0\\ 0 & {\left(q+rD\right)}^{2} \end{array}\right] \end{array}$$

We propose an experimental model based on target distance *D*, the Heisenberg effect, and visual error. Simultaneously, we explore the impact of four parameters on the endpoint distribution and predict the expectation µ and covariance matrix ∑ of the two-dimensional normal distribution through target distance.

### 2.2. Multi-Target Pointing Endpoint Distribution Model

Based on the derivation from the ST-PEDM, we propose a model for the endpoint distribution based on the distance between the pointing target and the interfering target and the direction of the interfering target.

In real-world scenarios, users typically do not separate selection confirmation from pointing tasks, leading to the Heisenberg effect during pointing tasks. Building upon Equation (4), we derive the endpoint distribution model for pointing towards targets with interference.

The distance between the interfering target and the actual selection target significantly influences the endpoint distribution. When the distance between them reaches a critical value, the impact of the interfering target on the endpoint distribution becomes negligible. For interfering targets at different azimuths, the endpoint distribution shifts in the opposite direction from the interfering target.

The theoretical foundation of multi-target and single-target pointing tasks is similar. Building upon Equation (4), we derive horizontal Equation (5) and vertical Equation (6) expressions for the positions and motion directions of horizontally and vertically interfering targets, respectively:  

(5) $$\left\{\begin{array}{cc} \; & \mu =\left[\begin{array}{c} a+bD\pm (c\frac{D}{{{(}_{e}/2)}^{2}}cos\theta +d)\\ e+fD \end{array}\right]\\ & \Sigma =\left[\begin{array}{cc} {\left(g+hD+\right)}^{2} & 0\\ 0 & {\left(i+jD\right)}^{2} \end{array}\right] \end{array}\right.$$

(6) $$\left\{\begin{array}{cc} \; & \mu =\left[\begin{array}{c} a+bD\\ c+dD\pm (e\frac{D}{{{(}_{e}/2)}^{2}}sin\theta +f) \end{array}\right]\\ & \Sigma =\left[\begin{array}{cc} {\left(g+hD\right)}^{2} & 0\\ 0 & {\left(i+jD\right)}^{2} \end{array}\right] \end{array}\right.$$

Integrating formulas for horizontal and vertical directions yields a model formula, as in Equation (7). For the distribution of pointing endpoints in multi-target scenarios.

(7) $$\left\{\begin{array}{cc} \; & \mu =\left[\begin{array}{c} a+bD\pm (c\frac{D}{{{(}_{e}/2)}^{2}}cos\theta +d)\\ e+fD\pm (g\frac{D}{{{(}_{e}/2)}^{2}}sin\theta +h) \end{array}\right]\\ & \Sigma =\left[\begin{array}{cc} {\left(i+jD\right)}^{2} & 0\\ 0 & {\left(k+lD\right)}^{2} \end{array}\right] \end{array}\right.$$

And, by extending Equation (7) to multiple interfering targets, we derive Equation (8). a,b,c,d,e,f,g,h,i,j,k,l,m,n are empirical constants, and  θ is the angle between the line connecting the pointing target and the interfering target and the horizontal axis. We propose an experimental model based on the distance and direction of interfering targets, simultaneously exploring the influence of two parameters on the endpoint distribution model. Additionally, we predict the expectation μ of the two-dimensional normal distribution through variations in target distance.

(8) $$\left\{\begin{array}{cc} \; & \mu =\left[\begin{array}{c} a+bD\pm P\pm e\frac{D}{D_{e}{\left(D_{e}/2\right)}^{2}}\cos\theta \pm \cdots \\ f+gD\pm Q\pm j\frac{D}{{\left(D_{e}/2\right)}^{2}}\sin\theta \pm \cdots \end{array}\right]\\ \; & P=c\left(c\frac{D}{{\left(D_{e}/2\right)}^{2}}\cos\theta +d\right)\\ \; & Q=h\frac{D}{{\left(D_{e}/2\right)}^{2}}\sin\theta +i\\ \; & \Sigma =\left[\begin{array}{cc} {\left(k+lD\right)}^{2} & 0\\ 0 & {\left(m+nD\right)}^{2} \end{array}\right] \end{array}\right.$$

### 2.3. Region Partitioning Algorithm

Based on the target distribution model of multi-target interference, two region partitioning algorithms, BRPA and ERPA, are proposed, respectively, for the single target direction selection scene and the complex multi-target selection scene. The area where the target is located is divided, and the user’s pointing and selection intention are judged. When the pointing endpoint falls in the divided area, the user is determined to establish interaction with the current device.

BRPA is suitable for the selection task of a simple scene with a single target. It is proposed based on the target endpoint distribution model without interference. When the coordinates of the pointing endpoint are in the region of the ST-PEDM, the decision interacts with the pointing target.

**Figure 2:**
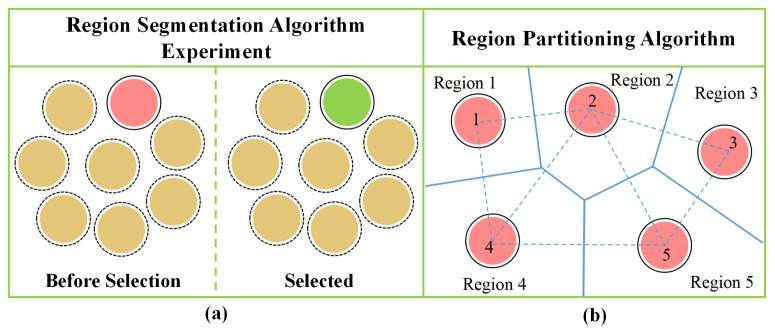
(**a**) Region segmentation algorithm experiment. Yellow circle: interference point; red circle: selected target; green circle: target after selection. (**b**) Region partitioning algorithm.

ERPA is suitable for the directional selection task in complex multi-target scenes, and is proposed based on the multi-interference target endpoint distribution model. First, the distance between the target and the interference target is calculated. When the distance is greater than 50 cm, as long as the coordinate of the pointing endpoint is in the region of the MT-PEDM, it is determined to interact with the pointing target. When the distance is less than 50 cm, the Voronoi diagram is used to divide the overlapping region again. The expected µx and µy on the *x* and *y* axes of the two overlapping regions are taken as vertices, and vertical bisecting lines are constructed to divide the overlapping region and form a continuous polygon region. It is implemented through Algorithm 1. In complex environments with multiple targets and interfering points, Voronoi diagrams provide a mathematically precise method for dividing regions, ensuring that each target point has its own dedicated region. As long as the coordinates pointing to the endpoint are within the final division area, the decision interacts with the pointing target, as shown in Figure 2b.
**Algorithm 1** Dividing regions and computing Voronoi diagram   1:**Input:**   2:    Target points $P=\{P_{1},P_{2},\cdots ,P_{n}\}$,   3:    Interfering target points $I=\{I_{1},I_{2},\cdots ,I_{n}\}$,   4:    Threshold distance = 50 cm   5:**Output:**   6:    Divided regions and Voronoi diagram for overlapping regions   7:**Procedure CALCULATE_DISTANCE**(p1, p2)   8:     Return the distance between points p1 and p2   9:**End Procedure**   10:**Procedure DIVIDE_REGIONS**()   11:    Initialize regions_list   12:    Initialize Voronoi_edges_list   13:**for** each target point pt in *P* **do**   14:    Initialize region for pt   15:    **for** each interfering target it in *I* **do**   16:        distance=CALCULATE_DISTANCE(pt,it)   17:        **if** distance≤50 **then**   18:            Add it to pt’s region   19:        **end if**   20:     **end for**   21:     Add pt’s region to regions_list   22:**end for**   23:**for** each pair of overlapping regions in regions_list **do**   24:     Add bisector based on $\mu_{x},\mu_{y}$   25:**end for**   26:Return divided regions and Voronoi diagram for overlapping regions   27:**End Procedure**

## 3. Experiment Task and Measurements

### 3.1. Participants

In this study, 15 participants were recruited, consisting of 11 males and four females. Two participants were left-handed, while thirteen were right-handed. The age range of the participants was from 23 to 28 years, with a median age of 24 years and an average age of 24.6 years. The participants had a minimum height of 164 cm, a maximum height of 191 cm, and an average height of 173.6 cm. All participants were in good health without disabilities and had experience in directional interactive operations. The experiment for each participant lasted approximately 2 h and collected a total of 31,050 experimental data points.

### 3.2. Apparatus

The experimental equipment used in this study mainly included one computer, one projector, and one set of motion capture equipment (including 12 high-speed infrared cameras, operating at 100 Hz frequency, with sub-millimeter accuracy). The computer was primarily used for calculating endpoint coordinates, creating the pointing feedback interface, monitoring the experiment’s runtime status in the background, developing the experiment program, and analyzing data post-experiment. The motion capture system served as the experimental environment, automatically recording experimental data such as time and the three-dimensional coordinates of various markers. The projector provided visual information feedback for participants during the experiment.

### 3.3. Experimental Design and Procedures

**Figure 3:**
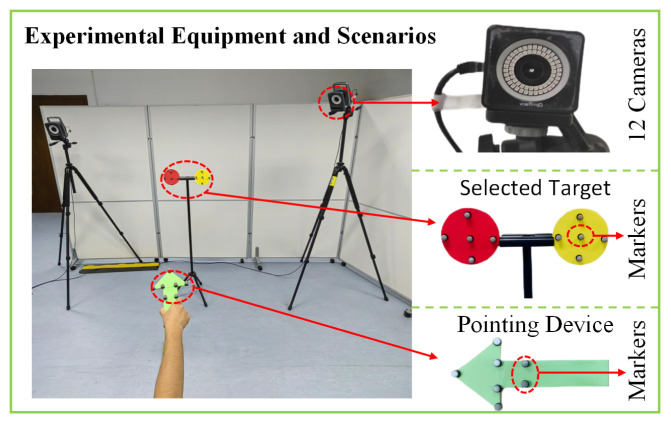
Experimental equipment and scenarios.

We designed a pointing device equipped with markers, along with several planar circles marked with reference points as selectable targets, as shown in Figure 3. First, the targets were placed within the range of the motion capture system, and their actual coordinates were determined based on the captured markers. Next, the experimenter held the pointing device and performed selection tasks at varying distances from the targets. Using the marker coordinates captured from the pointing device, an arrowed line was calculated to represent the virtual pointing trajectory. When the virtual ray intersected with a target, the target was considered successfully selected.

#### 3.3.1. ST-PEDM Experiment

The experiment was divided into three groups: a no-Heisenberg effect experiment, a Heisenberg effect experiment, and a control group experiment. The experiment’s independent variable was the distance between participants and the target (60 cm, 120 cm, 180 cm, 240 cm, and 300 cm). In the no-Heisenberg effect experiment, participants used their dominant hand to hold the indicator for pointing actions, followed by the other hand to grasp the mouse and click the right button for confirmation actions after pointing to the target. After confirmation, the indicator box changed from pink to light green, and each target distance was repeated 50 times.

Heisenberg effect experiment: Participants used their dominant hand to hold both the indicator and the mouse for pointing actions. After pointing to the target, they clicked the right mouse button to confirm the action. Upon confirmation, the indicator box changed from pink to light blue. This process was repeated 50 times for each target distance.

Control group experiment: To mitigate the potential influence of subtle differences in the experimental environment on the distribution of endpoints, which could impact data comparisons with the first two experimental groups, the control group experiment maintained the same experimental environment. Participants alternated between holding the mouse with their dominant and non-dominant hands to perform clicking confirmations. After confirmation, the indicator box changed from pink to light blue for the Heisenberg effect condition and from pink to green for the no Heisenberg effect condition. This process was repeated 20 times for each target distance.

The overall design of the experiment yielded a total of 15 participants × 5 target distances × 50 trials for the pointing task × 2 groups (Heisenberg and no Heisenberg) + 15 participants × 5 target distances × 20 trials for the pointing task × 1 group (control) = 9000 experimental trials.

#### 3.3.2. MT-PEDM Experiment

**Figure 4:**
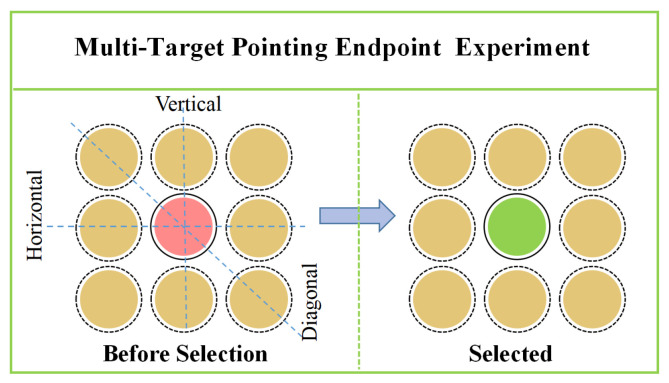
Multi-target pointing endpoint experiment. Yellow circle: interference point; red circle: selected target; green circle: target after selection.

The experiment was divided into three groups: Horizontal Placement of Interfering Objects Experiment, Vertical Placement of Interfering Objects Experiment, and Diagonal Placement of Interfering Objects (Control Group) Experiment, as shown in Figure 4. The distance between participants and the target was set at 180 cm. The independent variables of the experiment were the direction of interfering objects and the distance between the interfering objects and the target point (10 cm, 20 cm, 30 cm, 50 cm, and 75 cm).

Horizontal Placement of Interfering Objects Experiment: To investigate the impact of the orientation of target placement on the distribution of endpoint positions along the *x*-axis, two target points were placed horizontally. Participants alternately selected between the two target points and repeated the experiment 50 times. The distance of interfering points was adjusted for the next set of experiments.

Vertical Placement of Interfering Objects Experiment: To examine the influence of the orientation of target placement on the distribution of endpoint positions along the *y*-axis, two target points were placed vertically. Participants chose between the two target points alternately, and the experiment was repeated 50 times. The distance of interfering points was adjusted for the next set of experiments.

Diagonal Placement of Interfering Objects (Control Group) Experiment: To assess the reliability of the effects observed in the first two groups in a real-world scenario, considering the combined impact of horizontally and vertically placed interfering objects on endpoint distribution, interfering points were placed at a 45° angle relative to the target points. After participants completed 50 pointing tasks, the distance of interfering points was adjusted for the next set of experiments.

When participants engaged in the pointing task, the experimental interface indicated the target point that needed to be selected. Upon clicking the confirmation action, the target point indicator box in the experimental interface changed from pink to light green, signaling to the participants that they had completed the task, and data were recorded, as shown in Figure 4.

Before the experiment began, participants were provided with a detailed explanation of the experimental procedures and requirements. Additionally, a single training session was conducted. The overall design of the experiment resulted in 15 participants × 5 distances between interfering objects and target points × 50 trials for the pointing task × 3 groups = 11,250 experimental trials.

#### 3.3.3. Region Partitioning Algorithm Experiment

The design of the experimental task was similar to that of the ISO9241-9 [11] multi-directional tapping task; 8 target points were set, each target point was set to a fixed size, and the target point was uniformly placed on a circle. The participants completed the corresponding orientation selection task at three different target distances of 100 cm, 200 cm, and 300 cm.

When participants performed the directional selection task, the experimental interface indicated the target points to be selected; the target to be selected was light red, and the remaining seven target points were light yellow, as shown in Figure 2a.

Target selection completion time test experiment: When pointing to the endpoint and entering the target division area, the target point indicator box in the experiment interface changed from light red to light green, indicating that the participant had correctly selected the target and could click to confirm. Participants clicked the left mouse button, the timing ended, and the background recorded the selection time.

Pointing task accuracy test experiment: When participants thought they had pointed to the target, they clicked the left mouse button to confirm. Based on whether the pointing endpoint was in the target area, the background determined whether the pointing task was correct or wrong and recorded it.

Before the start of the experiment, the participants were informed about the experimental steps and requirements in detail, and 2 groups of training experiments were carried out. The design of the experiment yielded a total of 15 participants × 3 target distances × 8 targets × 5 times × 3 situations = 10,800 experiments.

## 4. Experimental Results

### 4.1. Results of the ST-PEDM

A normality test was conducted on five sets of data at different target distances using the Kolmogorov–Smirnov test. This was performed to ascertain the normal distribution on the *x*-axis and *y*-axis, with a confidence level of α=0.05. A Pearson correlation test was also employed to assess the correlation between *x* and *y*. A correlation coefficient 0 ± 0.3 is generally considered to indicate a weak or negligible correlation. The analysis results are presented in Table 1.

For the independent variable target distance, the expected values and standard deviations for the five groups of different data sets were calculated. Linear fitting was performed using Origin for their expected values and standard deviations on the *x*-axis and *y*-axis. We derive Equation (9).

(9) $$\left\{\begin{array}{cc} \; & \mu_{x}=-0.74+0.017D\;\;\;\;R^{2}=0.786\\ \; & \sigma_{x}=-0.41+0.034D\;\;\;\;R^{2}=0.992\\ \; & \mu_{y}=-5+0.15D\;\;\;\;\;\;\;\;R^{2}=0.984\\ \; & \sigma_{y}=-3.61+0.103D\;\;\;\;R^{2}=0.985 \end{array}\right.$$

For the independent variable target distance, the expected values and standard deviations for the five different data sets were calculated. Linear fitting was performed using Origin for their expected values and standard deviations on the *x*-axis and *y*-axis. We derive Equation (10). The results are presented in Table 2.

(10) $$\left\{\begin{array}{cc} \; & \mu_{x}=-0.82+0.028D\;\;\;\;R^{2}=0.907\\ \; & \sigma_{x}=-1.86+0.041D\;\;\;\;R^{2}=0.962\\ \; & \mu_{y}=-5+0.15D\;\;\;\;\;\;\;\;R^{2}=0.977\\ \; & \sigma_{y}=-4.96+0.124D\;\;\;\;R^{2}=0.97 \end{array}\right.$$

### 4.2. Results of the MT-PEDM

#### 4.2.1. The Impact of Horizontal Arrangement of Interfering Points on Endpoint Distribution

Pairwise comparison analysis was conducted for inter-group data differences, and the results are presented in Table 3. The analysis of the expected values for the *x*-axis coordinates of the endpoint distribution in the horizontal direction revealed a shift in the endpoint distribution opposite to the interfering targets.

Through inter-group analysis of the endpoint distribution coordinates (*x* and *y*) for interfering targets placed in different orientations, it is evident that the horizontal arrangement of interfering points significantly affects only the *x*-axis coordinates of the endpoint distribution. Simultaneously, the inter-group analysis of *x*-axis coordinates for different interfering target distances reveals a significant impact for the three groups within distances less than 50 cm. In other words, there is no significant impact on the endpoint coordinates for distances greater than or equal to 50 cm. The analysis of the expected values for the endpoint coordinates with interfering targets placed in different orientations indicates a varying degree of offset in the *x*-axis expectation within 30 cm, opposite the direction of the interfering object. For the independent variable of interfering target distance, the five groups’ data were linearly fitted using Origin to obtain the offset Δx on the *x*-axis. After adjustment, the fitted equation for the offset Δx yielded an $R^{2}$ of 0.979.

#### 4.2.2. The Impact of Vertically Arranged Interfering Points on Endpoint Distribution

A correction was applied using the Greenhouse–Geisser method ($F_{2.821,561.328}=4.652$, *p* = 0.004). This indicates a significant impact of different interfering target distances on the endpoint distribution in the *y*-axis direction during pointing tasks when interfering targets are placed above or below. Pairwise comparison analysis was conducted for inter-group data differences, and the results are presented in Table 4.

Through inter-group analysis of the endpoint distribution coordinates (*x* and *y*) for interfering targets placed in different vertical orientations, it is evident that the vertical arrangement of interfering points (above and below) significantly affects only the *y*-axis coordinates of the endpoint distribution, with no significant impact on the *x*-axis coordinates. Simultaneously, the inter-group analysis of *y*-axis coordinates for different interfering target distances reveals a significant impact for the three groups within distances less than 50 cm. In other words, there is no significant impact on the endpoint coordinates for distances greater than or equal to 50 cm. The analysis of the expected values for the endpoint coordinates with interfering targets placed in different vertical orientations indicates a varying degree of offset in the *y*-axis expectation within 30 cm, opposite the direction of the interfering object. For the independent variable of interfering target distance, the five groups’ data were used to calculate the offset Δy on the *y*-axis. Using Origin, linear fitting was applied to obtain the fitted equation for the offset Δy, with an adjusted goodness of fit $R^{2}$ of 0.957.

#### 4.2.3. The Impact of Interfering Target Orientation on Endpoint Distribution

A correction was applied using the Greenhouse–Geisser method ($F_{2.656,528.560}=12.913$, *p* < 0.01). This indicates a significant impact of different interfering target distances on the endpoint distribution in the *x*-axis direction during pointing tasks when interfering targets are placed in the upper-left and lower-right directions. Pairwise comparison analysis was conducted for inter-group data differences, and the results are presented in Table 5.

In the repeated measures of the analysis of variance for the *y*-axis coordinates when interfering targets were placed in the upper-left direction, the Mauchly test for interfering target distance resulted in $X^{2}\left(5\right)=28.507,\;p<0.001$, indicating a violation of the assumption of sphericity. A correction was applied using the Greenhouse–Geisser method ($F_{2.713,518.121}=14.781,\;p<0.01$). Similarly, in the repeated measures of the analysis of variance for the *y*-axis coordinates when interfering targets were placed in the lower-right direction, the Mauchly test for interfering target distance resulted in $X^{2}\left(5\right)=75.322,\;p<0.001$, indicating a violation of the assumption of sphericity. A correction was applied using the Greenhouse–Geisser method ($F_{2.433,484.25}=2.068$, *p* = 0.047). This indicates a significant impact of different interfering target distances on the endpoint distribution in the *y*-axis direction during pointing tasks when interfering targets are placed in the upper-right and lower-right directions. Pairwise comparison analysis was conducted for inter-group data differences, and the results are presented in Table 6.

#### 4.2.4. Fitting the Model

Through data analysis of the impact of interfering target distance and interfering target direction on the model and linear fitting of the expected impact offset values Δx and Δy, sbstituting the empirical constants in Equation (7), the model for the MT-PEDM under the Heisenberg effect was obtained, as shown in Equation (11).

(11) $$\left\{\begin{array}{cc} \; & \mu =\left[\begin{array}{c} -0.82\pm (0.67\frac{DA}{D_{e}}cos\theta +5.45)+0.028D\\ -5\pm (0.51\frac{DA}{D_{e}}sin\theta +13.96)+0.041D \end{array}\right]\\ & \Sigma =\left[\begin{array}{cc} {\left(-1.86+0.04D\right)}^{2} & 0\\ 0 & {\left(-4.96+0.12D\right)}^{2} \end{array}\right] \end{array}\right.$$

And, by extending Equation (11) to multiple interfering targets, the MT-PEDM was obtained, as shown in Equation (12).

(12) $$\left\{\begin{array}{cc} \; & \mu =\left[\begin{array}{c} -0.82+0.028D\pm P1\pm P2\pm \cdots \\ -5+0.041D\pm Q1\pm Q2\pm \cdots \end{array}\right]\\ \; & P1=0.67\frac{DA}{D_{\varepsilon }}\cos\theta +5.45\\ \; & P2=0.67\frac{DA}{D_{e}}\cos\theta \\ \; & Q1=0.51\frac{DA}{D_{s}}\sin\theta +13.96\\ \; & Q2=0.51\frac{DA}{D_{e}}\sin\theta \\ \; & \Sigma =\left[\begin{array}{cc} {\left(-1.86+0.04D\right)}^{2} & 0\\ 0 & {\left(-4.96+0.12D\right)}^{2} \end{array}\right] \end{array}\right.$$

### 4.3. The Results of the Region Partitioning Algorithm

**Figure 5:**
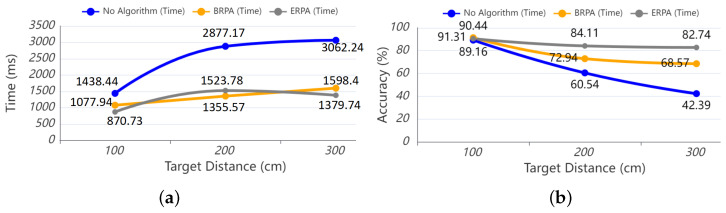
(**a**) Completion time of target selection using different regional partitioning algorithms; (**b**) accuracy of target selection using different regional partitioning algorithms.

In the target selection task with a target distance of 100 cm, the BRPA had a slight advantage over the ERPA, and the BRPA even took a shorter time to complete the target selection task than the ERPA. According to the data analysis, this is because the target distance of 100 cm allowed participants to select the target more accurately, and the interference target had less influence on the direction selection. In the target selection task with target distances of 200 cm and 300 cm, the BRPA and ERPA showed their advantages. Compared with the task completion time without the region partitioning algorithm, the task completion time of BRPA and ERPA was reduced by half or more, the data result is shown in Figure 5a. At the same time, the ERPA also completes the directional selection task faster than the BRPA.

In the target selection task with a target distance of 100 cm, there is almost no difference in the accuracy of the three region partitioning methods, and the accuracy of the three region partitioning algorithms reaches about 90%. It is also because, at the target distance of 100 cm, the participants can select the target more accurately, and the interference target has less influence on the direction selection. In the task of pointing target selection at target distances of 200 cm and 300 cm, the accuracy decreases to a certain extent with the increase of the distance, but the BRPA and the ERPA still show advantages. At the target distance of 200 cm, the BRPA obtains 72.94% accuracy. The accuracy of the ERPA is 84.11%, while the accuracy of the non-algorithm is only 60.54%. At the target distance of 300 cm, the accuracy of the BRPA is 68.57%, and the accuracy of the ERPA is 82.74%, the data result is shown in Figure 5b. The accuracy of the ERPA is also greatly improved compared with that of the BRPA.

## 5. Discussion and Conclusions

By investigating the impact of factors such as target distance, Heisenberg effect, visual error, hand tremor, and interfering target distance on endpoint distribution, we proposed ST-PEDM and MT-PEDM, and, based on these two models, we proposed BRPA and ERPA.

In the study of the directional endpoint distribution model, EDmodel has received positive feedback for pointing tasks in both AR and VR scenarios [7]. We will investigate whether EDmodel fits pointing tasks in real-world scenarios. EDmodel proposes the interaction effect of target distance and target width on the covariance matrix. We fitted our experimental data into the ED model, and the fitting results are presented in Table 7. The results indicate significant improvement in fitting our proposed ST-PEDM, making it more suitable for cursor-free pointing selection tasks in real-world scenarios.

Following a bivariate normal distribution, the endpoint distribution of single-target pointing tasks is determined by target distance and the Heisenberg effect. The distributions along the *x*-axis and *y*-axis are relatively independent. This model accurately describes the distribution area of endpoints in single-interaction target scenarios.

The endpoint distribution of multi-target pointing tasks is determined by target distance, the Heisenberg effect, distance to interfering targets, and their orientation. The offset in the mean of the endpoint distribution model is strongly correlated with the distance to interfering targets. This model provides a relatively accurate description of the distribution area of endpoints in complex scenarios with multiple targets.

Through data analysis of completion times for pointing selection tasks, it is evident that BRPA and ERPA significantly improve completion times for real-space cursor-free pointing selection tasks. Moreover, the advantages of these algorithms are more pronounced when the target distance is greater.

Although the model proposed in this paper achieved good fitting in experimental validation, there are still some limitations. The experimental model was conducted using the high-precision motion capture platform in a laboratory setting. In real-world scenarios, sensor accuracy is often limited by device size and portability constraints, making it difficult to match the precision of laboratory platforms. Indoor positioning and pointing angle determination typically rely on ultra-wideband technology, which inherently has sub-meter level distance errors and 15-degree angular errors, differing from the precision of the endpoint distribution model’s experimental platform.

For future work, we aim to validate the applicability of the proposed model and region partitioning algorithms by assembling a pointing selection platform that utilizes ultra-wideband technology. Additionally, we plan to apply the proposed model and algorithms to the rapid capture control and trajectory planning of drone swarms. This approach will enable precise and rapid targeting and control of target drones using pointing interaction technology, combined with smart glasses for command issuance and data transmission, thereby achieving drone control and trajectory planning.

## Figures and Tables

**Table 1:** Normality and correlation analysis results of endpoint distribution.

Target Distance (D)	60 cm	120 cm	180 cm	240 cm	300 cm
Normality Test *p*-Value X	0.49529	0.05093	0.73017	0.05113	0.0529
Normality Test *p*-Value Y	0.473	1	0.63041	0.10038	0.61323
Correlation Coefficient P	<0.001	<0.001	<0.001	0.29167	<0.001

**Table 2:** The results of the intrapersonal effect test between Heisenberg effect and target distance.

		Degrees of Freedom	F	*p*	Significance
Heisenberg Effect	X	1, 345	46.925	<0.001	YES
	Y	1, 503	5.227	0.023	YES
Target Distance	X	2.898, 999.726	188.726	<0.001	YES
	Y	2.515, 1265.2	637.417	<0.001	YES
Heisenberg Effect & Target Distance	X	2.482, 856.408	6.308	0.001	YES
	Y	2.241, 1127.412	6.367	0.001	YES

**Table 3:** Comparative analysis of differential *x*-coordinates at different interfering target distances.

*p* (α = 0.05)	Left					Right				
	10 cm	20 cm	30 cm	50 cm	75 cm	10 cm	20 cm	30 cm	50 cm	75 cm
10 cm	N.A.	<0.001	<0.001	0.001	<0.001	N.A.	<0.001	<0.001	<0.001	<0.001
20 cm	<0.001	N.A.	<0.001	<0.001	<0.001	<0.001	N.A.	<0.001	<0.001	<0.001
30 cm	<0.001	<0.001	N.A.	0.111	0.747	<0.001	<0.001	N.A.	0.143	0.531
50 cm	0.001	<0.001	0.111	N.A.	0.958	<0.001	<0.001	0.143	N.A.	0.751
75 cm	<0.001	<0.001	0.747	0.958	N.A.	<0.001	<0.001	0.531	0.751	N.A.

**Table 4:** Comparative analysis of differential *y*-coordinates at different interfering target distances.

*p* (α = 0.05)	Above					Below				
	10 cm	20 cm	30 cm	50 cm	75 cm	10 cm	20 cm	30 cm	50 cm	75 cm
10 cm	N.A.	<0.001	0.023	<0.001	<0.001	N.A.	0.006	0.001	0.021	<0.001
20 cm	<0.001	N.A.	<0.001	<0.001	<0.001	0.006	N.A.	<0.001	<0.001	<0.001
30 cm	0.023	<0.001	N.A.	0.514	013	0.001	<0.001	N.A.	0.391	0.31
50 cm	<0.001	<0.001	0.514	N.A.	0.9	0.021	<0.001	0.391	N.A.	0.962
75 cm	<0.001	<0.001	0.13	0.9	N.A.	<0.001	<0.001	0.31	0.962	N.A.

**Table 5:** Inter-group comparative analysis of *x*-coordinate differences at different distances of interfering targets under diagonal placement of interference points.

*p* (α = 0.05)	Top Left					Bottom Right				
	10 cm	20 cm	30 cm	50 cm	75 cm	10 cm	20 cm	30 cm	50 cm	75 cm
10 cm	N.A.	<0.001	<0.001	<0.001	0.021	N.A.	<0.001	<0.001	<0.001	<0.001
20 cm	<0.001	N.A.	<0.001	<0.001	<0.001	<0.001	N.A.	<0.001	<0.001	<0.001
30 cm	<0.001	<0.001	N.A.	0.507	0.217	<0.001	<0.001	N.A.	0.181	0.37
50 cm	<0.001	<0.001	0.507	N.A.	0.47	<0.001	<0.001	0.181	N.A.	0.264
75 cm	0.021	<0.001	0.217	0.47	N.A.	<0.001	<0.001	0.37	0.264	N.A.

**Table 6:** Inter-group comparative analysis of *y*-coordinate differences at different distances of interfering targets under diagonal placement of interference points.

*p* (α = 0.05)	Top Left					Bottom Right				
	10 cm	20 cm	30 cm	50 cm	75 cm	10 cm	20 cm	30 cm	50 cm	75 cm
10 cm	N.A.	<0.001	0.001	<0.001	<0.001	N.A.	<0.001	0.037	0.032	0.047
20 cm	<0.001	N.A.	0.001	<0.001	<0.001	<0.001	N.A.	<0.001	<0.001	0.001
30 cm	0.001	0.001	N.A.	0.229	0.139	0.037	<0.001	N.A.	0.433	0.985
50 cm	<0.001	<0.001	0.229	N.A.	0.157	0.032	<0.001	0.433	N.A.	0.63
75 cm	<0.001	<0.001	0.139	0.157	N.A.	0.047	0.001	0.985	0.63	N.A.

**Table 7:** Comparison of goodness of fit between EDmodel and ST-PEDM.

$R^{2}$	$\mu_{x}$	$\mu_{y}$	$\sigma_{x}$	$\sigma_{y}$
**EDmodel** $\mu =\left[\begin{array}{c} a+bD\\ 0 \end{array}\right]$ $\Sigma =\left[\begin{array}{cc} {P1\;}^{1} & 0\\ 0 & {p2\;}^{2} \end{array}\right]$	0.84	0	0.792	0.823
**ST-PEDM** $\mu =\left[\begin{array}{c} m+(b+n)D\\ c+dD \end{array}\right]$ $\Sigma =\left[\begin{array}{cc} {Q1\;}^{3} & 0\\ 0 & {Q2\;}^{4} \end{array}\right]$	0.907	0.977	0.962	0.97

^1^ $P1={\left(a+2\arcsin\left(W/D\right)\right)}^{2}$; ^2^ $P2={\left(c+2\arcsin\left(W/D\right)\right)}^{2}$; ^3^ $Q1={\left(o+pD\right)}^{2}$; ^4^ $Q2={\left(q+rD\right)}^{2}$.

## Data Availability

The research data are available upon request from the corresponding author.

## References

[1] Lindeman R.W., Sibert J.L., Hahn J.K. Hand-held windows: Towards effective 2D interaction in immersive virtual environments. Proceedings of the Proceedings IEEE Virtual Reality (Cat. No. 99CB36316).

[2] Veluvolu K.C., Ang W.T. (2011). Estimation of physiological tremor from accelerometers for real-time applications. Sensors.

[3] Laplante P. (1990). Heisenberg uncertainty. ACM SIGSOFT Softw. Eng. Notes.

[4] Wolf D., Gugenheimer J., Combosch M., Rukzio E. Understanding the heisenberg effect of spatial interaction: A selection induced error for spatially tracked input devices. Proceedings of the 2020 CHI Conference on Human Factors in Computing Systems.

[5] Bowman D., Wingrave C., Campbell J., Ly V. (2001). Using Pinch Gloves(tm) for Both Natural and Abstract Interaction Techniques in Virtual Environments. https://eprints.cs.vt.edu/archive/00000547/.

[6] Argelaguet F., Andujar C. (2013). A survey of 3D object selection techniques for virtual environments. Comput. Graph..

[7] LaViola J.J., Kruijff E., McMahan R.P., Bowman D., Poupyrev I.P. (2017). 3D User Interfaces: Theory and Practice.

[8] Henrikson R., Grossman T., Trowbridge S., Wigdor D., Benko H. (2020). Head-Coupled Kinematic Template Matching: A Prediction Model for Ray Pointing in VR. Proceedings of the 2020 CHI Conference on Human Factors in Computing Systems, CHI ’20.

[9] Dalsgaard T.S., Knibbe J., Bergström J. Modeling pointing for 3D target selection in VR. Proceedings of the 27th ACM Symposium on Virtual Reality Software and Technology.

[10] Jeong J.W., Jeong J.Y. (2024). Effect of Onset Position of Ray Casting in Virtual Reality. Proceedings of the Extended Abstracts of the CHI Conference on Human Factors in Computing Systems, CHI EA ’24.

[11] Yu D., Liang H.N., Lu X., Fan K., Ens B. (2019). Modeling endpoint distribution of pointing selection tasks in virtual reality environments. ACM Trans. Graph. (TOG).

[12] Dang N.T., Le H.H.L.H.H., Tavanti M. Visualization and interaction on flight trajectory in a 3d stereoscopic environment. Proceedings of the 22nd Digital Avionics Systems Conference, 2003. DASC’03.

[13] Debarba H.G., Grandi J.G., Maciel A., Nedel L., Boulic R. (2013). Disambiguation canvas: A precise selection technique for virtual environments. Human-Computer Interaction–INTERACT 2013, Proceedings of the 14th IFIP TC 13 International Conference, Cape Town, South Africa, 2–6 September 2013.

[14] Zhao C., Li K.W., Peng L. (2023). Movement time for pointing tasks in real and augmented reality environments. Appl. Sci..

[15] Jota R., Nacenta M.A., Jorge J.A., Carpendale S., Greenberg S. A comparison of ray pointing techniques for very large displays. Proceedings of the Graphics Interface.

[16] Kohli L., Whitton M. (2005). The haptic hand: Providing user interface feedback with the non-dominant hand in virtual environments. Proceedings of the Graphics Interface 2005.

[17] König W.A., Gerken J., Dierdorf S., Reiterer H. (2009). Adaptive pointing–design and evaluation of a precision enhancing technique for absolute pointing devices. Human-Computer Interaction–INTERACT 2009, Proceedings of the 12th IFIP TC 13 International Conference, Uppsala, Sweden, 24–28 August 2009.

[18] Mäkelä V., Heimonen T., Turunen M. Magnetic cursor: Improving target selection in freehand pointing interfaces. Proceedings of the International Symposium on Pervasive Displays.

[19] Mayer S., Schwind V., Schweigert R., Henze N. The effect of offset correction and cursor on mid-air pointing in real and virtual environments. Proceedings of the 2018 CHI Conference on Human Factors in Computing Systems.

[20] Feiner A.O.S. The flexible pointer: An interaction technique for selection in augmented and virtual reality. Proceedings of the UIST.

[21] Murata A. (1999). Extending effective target width in Fitts’ law to a two-dimensional pointing task. Int. J. Hum.-Comput. Interact..

[22] Grossman T., Balakrishnan R. (2005). A probabilistic approach to modeling two-dimensional pointing. ACM Trans. Comput.-Hum. Interact. (TOCHI).

[23] Bi X., Li Y., Zhai S. FFitts law: Modeling finger touch with fitts’ law. Proceedings of the SIGCHI Conference on Human Factors in Computing Systems.

[24] Wei Y., Shi R., Yu D., Wang Y., Li Y., Yu L., Liang H.N. (2023). Predicting Gaze-based Target Selection in Augmented Reality Headsets based on Eye and Head Endpoint Distributions. Proceedings of the 2023 CHI Conference on Human Factors in Computing Systems, CHI ’23.

[25] Achanta R., Estrada F., Wils P., Süsstrunk S. (2008). Salient region detection and segmentation. Computer Vision Systems, Proceedings of the 6th International Conference, ICVS 2008, Santorini, Greece, 12–15 May 2008.

[26] Li S., Yang B. (2008). Multifocus image fusion using region segmentation and spatial frequency. Image Vis. Comput..

[27] Wan S., Zhao Y., Wang T., Gu Z., Abbasi Q.H., Choo K.K.R. (2019). Multi-dimensional data indexing and range query processing via Voronoi diagram for internet of things. Future Gener. Comput. Syst..

[28] Tang X., Tan L., Hussain A., Wang M. (2019). Three-dimensional Voronoi diagram–based self-deployment algorithm in IoT sensor networks. Ann. Telecommun..

[29] Masehian E., Amin-Naseri M. (2004). A voronoi diagram-visibility graph-potential field compound algorithm for robot path planning. J. Robot. Syst..

[30] Aurenhammer F. (1991). Voronoi diagrams—A survey of a fundamental geometric data structure. ACM Comput. Surv. (CSUR).

[31] Sugihara K., Iri M. (1992). Construction of the Voronoi diagram for’one million’generators in single-precision arithmetic. Proc. IEEE.

[32] Cao K., Chen Y.Q., Gao S., Yan K., Zhang J., An D. (2023). Omni-Directional Capture for Multi-Drone Based on 3D-Voronoi Tessellation. Drones.

[33] Latombe J.C. (2012). Robot Motion Planning.

[34] Bhattacharya P., Gavrilova M.L. (2008). Roadmap-based path planning-using the voronoi diagram for a clearance-based shortest path. IEEE Robot. Autom. Mag..

[35] Akyildiz I.F., Su W., Sankarasubramaniam Y., Cayirci E. (2002). Wireless sensor networks: A survey. Comput. Netw..

[36] Miah M.S., Knoll J. (2018). Area coverage optimization using heterogeneous robots: Algorithm and implementation. IEEE Trans. Instrum. Meas..

[37] Li B., Meng H., Zhu Y., Song R., Cui M., Chen G., Huang K. (2021). Enhancing 3-D LiDAR point clouds with event-based camera. IEEE Trans. Instrum. Meas..

[38] Li C., Chen X., Chai L. (2023). Simultaneous Coverage and Mapping of Stereo Camera Network for Unknown Deformable Object. IEEE Trans. Instrum. Meas..

[39] Afghantoloee A., Abolfazl Mostafavi M. (2024). A Purpose-Oriented 3-D Voronoi Algorithm for Deployment of a Multitype Sensor Network in Complex 3-D Indoor Environments in Support of the Mobility of People With Motor Disabilities. IEEE Trans. Instrum. Meas..

[40] Scott MacKenzie I. (2015). Fitts’ throughput and the remarkable case of touch-based target selection. Human-Computer Interaction: Interaction Technologies, Proceedings of the 17th International Conference, HCI International 2015, Los Angeles, CA, USA, 2–7 August 2015.

